# Comparative Study on the Antivirus Activity of Shuang–Huang–Lian Injectable Powder and Its Bioactive Compound Mixture against Human Adenovirus III In Vitro

**DOI:** 10.3390/v9040079

**Published:** 2017-04-12

**Authors:** Qinhai Ma, Dedong Liang, Shuai Song, Qintian Yu, Chunyu Shi, Xuefeng Xing, Jia-Bo Luo

**Affiliations:** 1 School of Traditional Chinese Medical Science, Southern Medical University, Guangzhou 510515, China; 13268268214@163.com (Q.M.); famouscool@163.com (D.L.); ss89112@126.com (S.S.); dkdklm@163.com (Q.Y.); 15626452676@163.com (C.S.); 2 Guangdong Provincial Key Laboratory of Chinese Medicine Pharmaceutics, Southern Medical University, Guangzhou 510515, China

**Keywords:** human adenovirus III, anti-viral, Shuang–Huang–Lian injectable powder, effect comparison

## Abstract

Shuang–Huang–Lian injectable powder (SHL)—a classical purified herbal preparation extracted from *Scutellaria baicalensis*, *Lonicera japonica*, and *Forsythia suspense*—has been used against human adenovirus III (HAdV_3_) for many years. The combination herb and its major bioactive compounds, including chlorogenic acid, baicalin, and forsythia glycosides A, are effective inhibitors of the virus. However, no comprehensive studies are available on the antiviral effects of SHL against HAdV_3_. Moreover, it remains unclear whether the mixture of chlorogenic acid, baicalin, and forsythia glycosides A (CBF) has enhanced antiviral activity compared with SHL. Therefore, a comparative study was performed to investigate the combination which is promising for further antiviral drug development. To evaluate their antivirus activity in parallel, the combination ratio and dose of CBF were controlled and consistent with SHL. First, the fingerprint and the ratio of CBF in SHL were determined by high performance liquid chromatography. Then, a plaque reduction assay, reverse transcription polymerase chain reaction (PCR), real-time polymerase chain reaction (qPCR), and enzyme-linked immunosorbent assay (ELISA) were used to explore its therapeutic effects on viral infection and replication, respectively. The results showed that SHL and CBF inhibited dose- and time-dependently HAdV_3_-induced plaque formation in A549 and HEp-2 cells. SHL was more effective than CBF when supplemented prior to and after viral inoculation. SHL prevented viral attachment, internalization, and replication at high concentration and decreased viral levels within and out of cells at non-toxic concentrations in both cell types. Moreover, the expression of tumor necrosis factor alpha (TNF)-α, interleukin (IL)-1ß, and IL-6 was lower and the expression of interferon (IFN)-γ was higher in both cell types treated with SHL than with CBF. In conclusion, SHL is much more effective and slightly less toxic than CBF.

## 1. Introduction

Human adenovirus (HAdV), a nonenveloped DNA virus, is a common causative pathogen of acute respiratory infection. HAdV infection is more common in childcare and overcrowded conditions, and at least one strain was detected in most infected children during the first 5 years after birth. Nowadays, seven species, including many genotypes, have been characterized by genomics and bioinformatics [1,2]. HAdV species B (serotypes 3, 7, 14, and 55), species C (serotypes 1, 2, 5, and 6), and species E (serotype 4) are the most commonly found in patients with respiratory infection. Among these, HAdV_3_ strains of subspecies B1 are the major epidemic strains responsible for severe respiratory disease epidemics and outbreaks worldwide [3,4,5,6,7,8,9,10]. Currently, there is no effective treatment or vaccine against HAdV_3_ infection and new anti-HAdV_3_ drugs urgently need to be developed.

Shuang–Huang–Lian injectable powder (SHL), consisting of *Scutellaria baicalensis, Lonicera japonica*, and *Forsythia suspense*, is a classical prescription in traditional Chinese medicine (TCM). Its efficacy against many infectious diseases caused by bacteria and viruses in respiratory traction has been demonstrated [11]. In 1992, SHL was approved in China as a new Chinese patent drug for emergency treatment. Chlorogenic acid, baicalin, and forsythia glycosides, the main effective compounds isolated from the herb extract, are representative markers of its quality recorded in the Chinese Pharmacopoeia (2015). Chlorogenic acid is the major ingredient of *Lonicera japonica* that has been reported to have multi-anti-viral activities against human immunodeficiency virus (HIV), adenovirus, influenza virus (H1N1, H5N1), and EV71 [12,13,14,15,16]. Baicalin, which is derived from the dried root of *Scutellaria baicalensis*, has been demonstrated to inhibit influenza A (H1N1) infection, Dengue virus, and respiratory syncytial virus infection [17,18,19]. Forsythoside A, the active ingredient of *Forsythia suspense*, has been shown to inhibit syncytial virus and coxsackievirus in vitro [20]. In addition, forsythoside A has the potential to prevent infectious bronchitis virus (IBV) infection in vitro [21].

Multidrug combination is an important strategy in modern antivirus therapy because of increasing drug resistance. Contrary to single-component drugs, the benefits of TCM drugs are often due to the synergistic interactions of multiple ingredients. Therefore, we hypothesized that the antivirus ability of SHL may occur though the activity of multiple ingredients with multiple targets. Accordingly, a comparative antivirus study of SHL and its major bioactive ingredients mixture was investigated. Antivirus effects, interference link, and regulation of inflammatory cytokines were explored in both HEp-2 and A549 cell lines.

## 2. Materials and Methods

### 2.1. Reagents

SHL (lot#: 1409422) was provided by Second Chinese Medicine Factory of Harbin Pharm Group Co., Ltd. (Harbin, China). Forsythoside A (94.1%, lot#: 111810–201405), baicalin (93.3%, lot#: 110715–201318), and chlorogenic acid (96.2%, lot#: 110753–201415) were provided by the National Institutes for Food and Drug Control (Beijing, China). Mouse tumor necrosis factor alpha (TNF-α), interleukin (IL)-1ß, IL-6, and interferon gamma (IFN-γ) enzyme-linked immunosorbent assay (ELISA) kits were obtained from Huamei (Wuhang, China). Premix Taq^TM^ (TaKaRa Taq^TM^ Version 2.0) and SYBR Premix Ex Taq II (Tli RNaseH Plus) were purchased from Takara (Tokyo, Japan). The virus DNA extraction kit was obtained from Guangzhou Institute of Respiratory Disease (Guangzhou developed by Pharmaceutical Technology Co., Ltd., Guangzhou, China). Cell counting kit-8 (CCK-8) was from Dojindo (Dojindo, Japan).

### 2.2. Chromatographic Conditions and Test Samples Preparation

**Figure 1:**
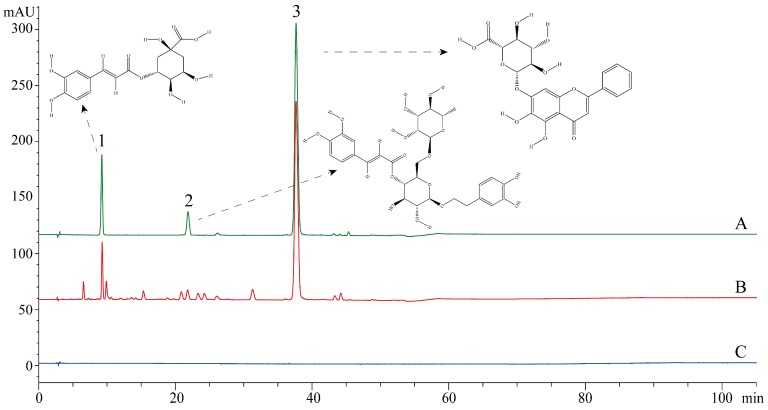
HPLC chromatograms of three effective constituents in SHL at 327 nm. 1. chlorogenic acid; 2. forsythoside A; 3. Baicalin.

The high performance liquid chromatography (HPLC)-diode array detector (DAD) fingerprint of SHL was performed on an Agilent 1200 series liquid chromatography system (Agilent Technologies, Santa Clara, CA, USA), consisting of a binary pump (Agilent G1312B), an auto-sampler (Agilent G1329B), and a DAD-vis detector (Agilent G1316A). The data were recorded and analyzed using Agilent Chemstation software for the LC-3D system (Rev. B.04.03-SP1). A Cosmosil 5C18-AR-II column (5 μm, 4.6 × 250 mm; Nacalai Tesque Co. Inc., Tokyo, Japan) was used at 30 °C. The mobile phase was composed of (A) phosphoric acid aqueous solution (0.2%, *v/v*) and (B) methanol using a gradient elution of 80–70% (A) at 0–5 min; 70–55% (A) at 5–35 min; and 55–30% (A) at 35–50 min. The sample injection volume was 10 μL with a flow rate of 1.0 mL/min. The detection wavelength was set at 327 nm. Three standard references, which were chlorogenic acid, forsythoside A, and baicalin, were used. The stock solutions of standard references were prepared by dissolving accurately the weighed standards in methanol and storing them in a 10 mL volumetric flask. Otherwise, 20.0 mg SHL was supplemented with 10 mL methanol and then extracted under sonication for 20 min. All the test samples were filtered through a 0.45 μm membrane filter before chromatographic analysis. The HPLC-DAD fingerprint standard of SHL was recorded and compared to these three standard references. HPLC-DAD revealed that SHL contained 1.3% chlorogenic acid, 0.8% forsythoside A, and 21.6% baicalein (Figure 1). Based on the ratios of the three main components of SHL measured by HPLC-DAD, we prepared a mixture of the three main components. Then, the antiviral effect of the mixed standards was compared with that of SHL. The initial concentration of the mixture was 593.3 μg/mL (the rate of the mixture of chlorogenic acid, forsythoside A, and baicalin was 1.3:0.8:21.6). The initial concentration of SHL was 20 mg/mL. The standard mix (including baicalin, chlorogenic acid, and forsythoside A) was prepared according to the composition ratio of SHL. Three chemical standards were weighed accurately and dissolved in Dulbecco’s Modified Eagle’s Medium (DMEM, Gibco).

### 2.3. Cells and Virus

HEp-2 cells (ATCC, Manassas, VA, USA) and A549 cells (ATCC, Manassas, VA, USA) were inoculated with HAdV3 (HAdV_3_, the Chinese Academy of Sciences Wuhan Institute of Virology, Wuhan, China). Cells were propagated at 37 °C under 5% CO_2_ in DMEM (Gibco, Carlsbad, CA, USA) cultured with 10% fetal bovine serum (FBS, Gibco) and 1% antibiotics (Gibco). Two percent FBS, instead of 10%, was used to propagate virus-infected cell monolayers. The virus was stored at −80 °C, and its titer was determined by 50% tissue culture infection dose (TCID_50_).

### 2.4. Cytotoxicity Assay

To determine whether SHL and CBF were toxic to HEp-2 and A549 cells, a cytotoxicity assay was measured using the cell counting kit-8 (CCK-8) assay [22]. Their 50% cytotoxic concentrations (CC_50_) were determined by plotting the percentage of cell growth inhibition against the concentration of the compound drug.

### 2.5. Antiviral Effect Assay

The antiviral activity of SHL and CBF was examined by plaque reduction assay, as previously described [23]. Briefly, 2 × 10^4^ cells/well were plated in 24-well culture plates at 37 °C under 5% CO_2_ for 24 h and inoculated with a mixture of 100 TCID_50_/well virus and various concentrations of SHL and CBF in triplicate at room temperature for 2 h. After supplementation with overlay medium (DMEM plus 2% FBS in 1% methylcellulose), they were cultured at 37 °C under 5% CO_2_ for 3 days. The monolayer was then fixed with 10% formalin and stained with 1% crystal violet, and then the plaques were counted. The minimal concentration of drugs required to inhibit 50% of the cytopathic effect (50% inhibitory concentration [IC_50_]) was calculated by the regression analysis of the dose–response curve generated from the data.

### 2.6. Time of Addition Assay

The antiviral activity of SHL and CBF was examined at different time points prior to and after viral inoculation by the plaque reduction assay [23]. Cells were seeded and incubated for 24 h as previously described. Various concentrations of SHL and the mixture were supplemented at 2 h (−2 h) or 1 h (−1 h) prior to viral inoculation, or 1 h (+1 h) or 2 h (+2 h) after viral inoculation. Supernatants were removed prior to the supplementation of overlay medium. Cells were incubated for an additional 72 h and examined by plaque assay.

### 2.7. Attachment Assay

Plaque reduction assay was performed to evaluate the effect of SHL and CBF on viral attachment [23]. Briefly, cells were seeded and incubated for 24 h. Cells were pre-chilled at 4 °C for 1 h, and the medium was replaced by a mixture of 100 TCID_50_/well virus and various concentrations of SHL and the CBF mixture. After incubation at 4 °C for another 3 h, the free virus was removed. The cell monolayer was washed with ice-cold phosphate-buffered saline (PBS) three times, covered with overlay medium, incubated at 37 °C under 5% CO_2_ for an additional 72 h, and examined by plaque assay.

### 2.8. Internalization Assay

The effect of SHL and CBF on viral internalization was also evaluated [23]. Briefly, cells were seeded and incubated for 24 h and pre-chilled at 4 °C for 1 h. Cells were infected with 100 TCID_50_/well virus and incubated at 4 °C for another 3 h. The virus-containing medium was replaced by fresh medium containing various concentrations of SHL and CBF in triplicate. They were shifted to culture at 37 °C. At 20 min, 40 min, and 60 min intervals following the 37 °C shift, un-internalized virus was inactivated by supplementation with acidic PBS (pH 3) for 1 min, followed by alkaline PBS (pH 11) for neutralization. Then, PBS was replaced by fresh overlay medium. After incubation at 37 °C for an additional 72 h, the cell monolayer was examined by plaque assay.

### 2.9. Polymerase Chain Reaction (PCR) and Quantitative PCR (qPCR)

The antiviral activity of SHL and CBF against viral replication was further examined by PCR semi-quantitatively and by qPCR quantitatively. Briefly, 4 × 10 ^5^ cells/well were plated into 6-well culture plates for 24 h. A mixture of 100 TCID_50_/well virus and various concentrations of SHL and CBF were supplemented. They were cultured for a further 48 h. Viral DNA was extracted with the virus DNA extraction kit for virus-infected A549 cells, HEp-2 cells, and culture supernatant, according to the manufacturer’s instruction. They were then placed on ice or at 4 °C for PCR and qPCR.

The PCR and qPCR sample systems and conditions were determined according to the instruction of Premix TaqTM (TaKaRa TaqTM Version 2.0) and SYBR Premix Ex Taq II (Tli RNaseH Plus), respectively. Amplification products were analyzed semi-quantitatively by 2% agarose gel electrophoresis, and the qPCR was detected by the Step One Real-Time PCR System (Mx3005P, Stratagene, La, Jolla, CA, USA). The forward primer of Adv3 was 5’-ATCGATGATGCCCCAATGG-3’, and the reverse primer was 5’-GGACTCAGGTACTCCGAAGCA-3’. Taking the CT value (cycle threshold) for the vertical axis and the copy number of the logarithm of concentration as abscissa, the standard curve was automatically generated by the qPCR instrument control software. The amount of virus of the experimental groups was calculated from the differences between the CT of the viral control and those of the experimental groups.

### 2.10. Enzyme-Linked Immunosorbent Assay (ELISA)

After the antiviral effect assays were performed, the culture medium was collected and assayed using the TNF-a, IFN-γ, IL-1ß, and IL-6 ELISA kit according to the manufacturer’s instructions. The A450 nm was determined by the ELISA reader (Thermo Scientific, Boston, MA, USA).

### 2.11. Statistical Analysis

Results are expressed as mean ± standard deviation (S.D.). Percentage of control (infection rate; %) was calculated from the plaque counts of the experimental groups divided by that of the viral control. Data were analyzed with analysis of variance (ANOVA) by SPSS ver. 19.0 (Armonk, NY, USA). Tukey honestly significant difference (HSD) test was used for post-hoc ANOVA comparisons. *p* < 0.05 was considered statistically significant.

## 3. Results

### 3.1. Cytotoxicity of SHL and CBF in A549 and HEp-2 Cells

**Figure 2:**
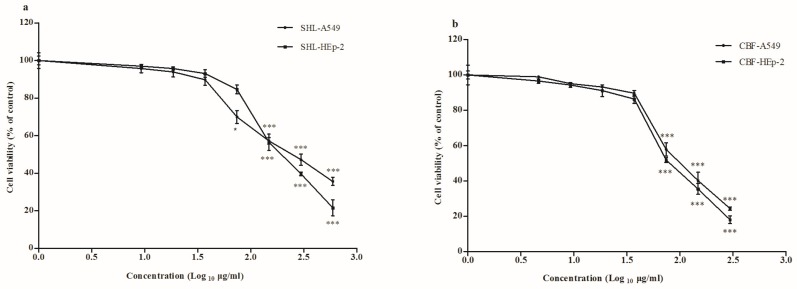
Shuang–Huang–Lian injectable powder (SHL) showed its cytotoxicity against host cells above the concentrations of 74.2 μg/mL on the A549 cells and above the concentrations of 148.3 μg/mL on the HEp-2 cells (*p* < 0.05) (**a**); chlorogenic acid, baicalin, and forsythia glycosides A (CBF) showed cytotoxicity above the concentrations of 74.2 μg/mL on both cells (*p* < 0.001) (**b**). Data are represented as mean ± S.D. of nine tests. * *p* < 0.05; ** *p* < 0.01; *** *p* < 0.001 were compared to the cell control.

To determine whether SHL and CBF were toxic to cells, a cytotoxicity assay was performed using the CCK-8 assay. The estimated CC_50_ of SHL and CBF were 297.7 and 148.2 μg/mL on A549 cells and 211.7 and 94.1 μg/mL on HEp-2 cells, respectively (Figure 2). Neither SHL treatment nor CBF treatment were significantly cytotoxic from concentrations of 37.1 μg/mL in the two cell types. Therefore, SHL and CBF at the concentrations of 37.1–1.2 μg/mL were selected in the subsequent experiments.

### 3.2. SHL Attenuated Virus Proliferation More Significantly Than CBF

**Figure 3:**
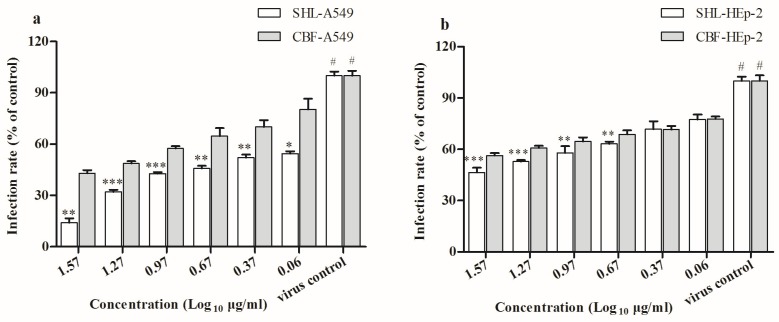
SHL and CBF were dose-dependently effective against human adenovirus III (HAdV_3)_ in both cell types as determined by plaque reduction assay (*p* < 0.05); SHL decreased more plaque formation than CBF at all the concentrations (*p* < 0.05) in A549 cells (**a**) and at the higher concentrations than 4.6 μg/mL (*p* < 0.01) in HEp-2 cells (**b**). Data are represented as mean ± S.D. of nine tests. * *p* < 0.05; ** *p* < 0.01; *** *p* < 0.001 were compared to CBF. # *p* < 0.05 was compared to the virus control.

SHL and CBF dose-dependently decreased virus proliferation in HEp-2 and A549 cells. The effect of SHL, however, showed better suppression than CBF in both HEp-2 cells and A549 cells (*p* < 0.05) (Figure 3). This effect was significantly different at all concentrations except for 2.3 and 1.2 μg/mL on Hep-2 cells (*p* < 0.05). It indicated that SHL was more effective at inhibiting the proliferation of the virus than that by CBF.

### 3.3. SHL Decreased Plaque Formation More Than CBF When Viral Inoculation Was Given in Different Working Points

**Figure 4:**
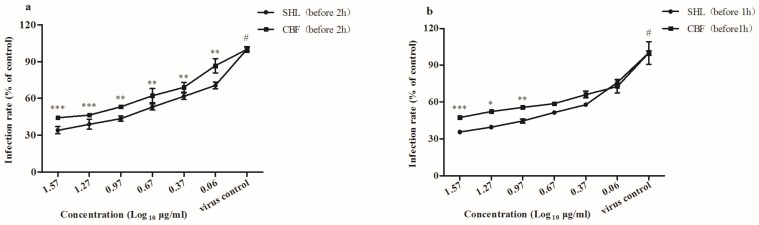

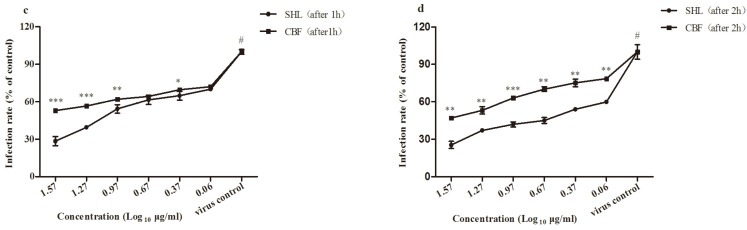
SHL and CBF were time-dependently and dose-dependently effective against HAdV_3_ when given viral inoculation in different administrations (*p* < 0.05), and SHL decreased more plaque formation than CBF in both cell types (*p* < 0.05). Data are represented as mean ± S.D. of nine tests. * *p* < 0.05; ** *p* < 0.01; *** *p* < 0.001 were compared to CBF. # *p* < 0.05 was compared to the virus control.

To better understand the therapeutic intervention during virus invasion, time of addition assay in A549 and HEp-2 cells was employed to explore its working points. SHL and CBF time-dependently and dose-dependently decreased plaque formation in A549 and HEp-2 cells. SHL decreased plaque formation more than CBF when viral inoculation was given in different working points (*p* < 0.05) (Figure 4). It showed that both SHL and CBF were better at inhibiting virus activity when given before viral inoculation than after in the two cells types. As the exposure duration of cells to SHL and CBF before viral inoculation increased, so did the significance of the antiviral activity.

### 3.4. SHL Inhibited Viral Attachment Better Than CBF in A549 and HEp-2 Cells

**Figure 5:**
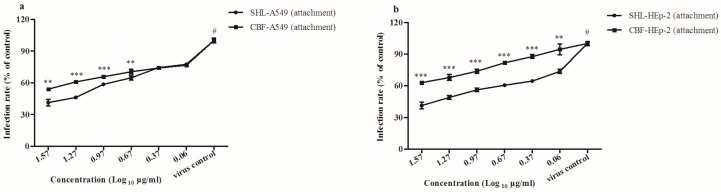
SHL and CBF were dose-dependently effective against viral attachment in both cell types (*p* < 0.05). SHL decreased more plaque formation than CBF at the higher concentration than 4.6 μg/mL in A549 cells (*p* < 0.05) (**a**), and at all the concentrations in HEp-2 cells (*p* < 0.05) (**b**). Data are represented as mean ± S.D. of nine tests. * *p* < 0.05; ** *p* < 0.01; *** *p* < 0.001 were compared to CBF. # *p* < 0.05 was compared to the virus control.

Because SHL and CBF anti-virus activity was mainly effective by supplementation before viral inoculation, we predicted that they worked by disrupting viral attachment and that the anti-viral effect of SHL was superior to that of CBF. Results from the attachment assay confirmed this hypothesis, as both SHL and CBF dose-dependently inhibited viral attachment. SHL decreased plaque formation more than CBF at concentrations higher than 4.6 μg/mL in A549 cells (*p* < 0.01) (Figure 5a), and SHL decreased plaque formation more than CBF at all concentrations in HEp-2 cells (*p* < 0.01) (Figure 5b). These results were consistent with those of the anti-viral effect assay (Figure 3) and the time course assay (Figure 4). It demonstrated that viral attachment was inhibited more with SHL than with CBF, and the effect was not significantly different between A549 and HEp-2 cells.

### 3.5. SHL Affected Viral Internalization More Than CBF

**Figure 6:**
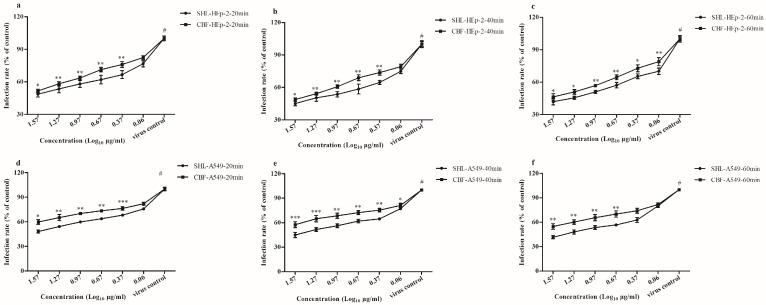
SHL and CBF were time-dependently and dose-dependently effective against viral internalization in both cell types (*p* < 0.05). SHL decreased more plaque formation than CBF at the high concentration (*p* < 0.05). Data are represented as mean ± S.D. of nine tests. * *p* < 0.05; ** *p* < 0.01; *** *p* < 0.001.

The results of the internalization assay (Figure 6) were consistent with those of the above assays (Figure 4). SHL decreased plaque formation more than CBF at all concentrations except for 1.2 μg/mL in A549 cells (20 min) (*p* < 0.05) (Figure 6a); at all the concentrations in A549 cells (40 min) (*p* < 0.05) (Figure 6b); and at concentrations higher than 4.6 μg/mL in A549 cells (60 min) (*p* < 0.01) (Figure 6c). In addition, SHL decreased plaque formation more than CBF at concentrations higher than 2.3 μg/mL in HEp-2 cells (20 min) (*p* < 0.05) (Figure 6d); at concentrations higher than 2.3 μg/mL in HEp-2 cells (40 min) (*p* < 0.05) (Figure 6e); and at all concentrations in HEp-2 cells (60 min) (*p* < 0.05) (Figure 6f). It indicated that the antiviral effects enhanced with increase in time.

### 3.6. SHL Significantly Suppressed Viral DNA Replication in A549 and HEp-2 Cells Compared to CBF

**Figure 7:**
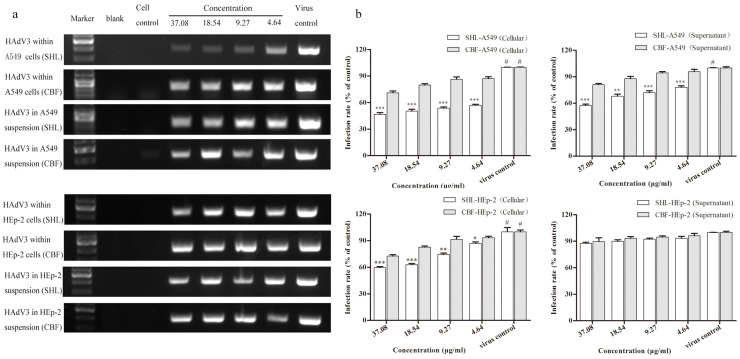
The PCR result of the quantification of viral DNA by the agarose gel electrophoresis (**a**) was comparable to that of the qPCR (**b**). SHL markedly decreased the viral amounts more than CBF at all concentrations (*p* < 0.001) within A549 cells (*p* < 0.001) and in the suspension (*p* < 0.05); and at all the concentrations within HEp-2 cells (*p* < 0.05); and there was no significant difference in the suspension (*p* > 0.05). Data are represented as mean ± S.D. of three tests. * *p* < 0.05; ** *p* < 0.01; *** *p* < 0.001 were compared to CBF. # *p* < 0.05 was compared to the virus control.

Data from the semi-quantification of viral DNA by agarose gel electrophoresis were comparable to those of qPCR (Figure 7). The results of the semi-quantification and quantification of viral DNA had different trends in cells and in the suspension. SHL markedly decreased viral amounts compared to CBF at all concentrations (*p* < 0.001) in A549 cells and at concentrations higher than 4.6 μg/mL (*p* < 0.01) in the suspension. In addition, SHL markedly decreased the amount of virus compared to CBF at concentrations higher than 4.6 μg/mL (*p* < 0.05) in HEp-2 cells. The difference, however, was not significant in the suspension.

### 3.7. SHL Attenuated Inflammatory Effects Significantly More Than CBF in HAdV_3_-Stimulated A549 and HEp-2 Cells

**Figure 8:**
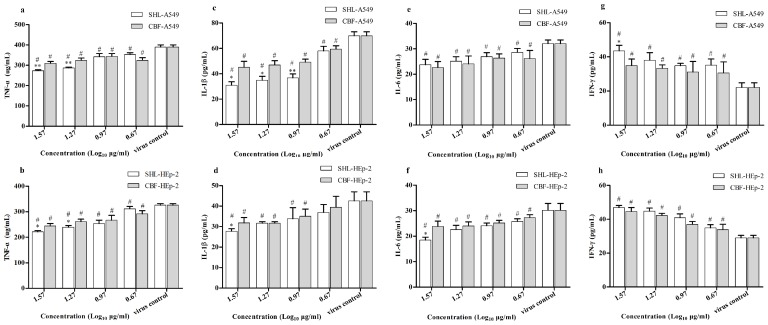
The expressions of TNF-α, IL-1ß, and IL-6 were lower and the expression of IFN-γ was higher in both cell types treated with SHL than with CBF. Data are represented as mean ± S.D. of three tests. * *p* < 0.05; ** *p* < 0.01; *** *p* < 0.001 were compared to CBF. # *p* < 0.05 was compared to the virus control.

Because virus infection can lead to the production of the pro-inflammatory cytokines IFN-γ, TNF-α, IL-1ß, and IL-6, which contribute to inflammation, the expressions of these cytokines were measured. Results showed that SHL and CBF significantly suppressed the secretion of these cytokines in both cell types compared to the virus control group (Figure 8). SHL, however, was more effective than CBF at decreasing the secretion of TNF-α at the concentrations of 37.1 and 18.5 μg/mL in both A549 and HEp-2 cells (Figure 8a,b). In addition, SHL suppressed the secretion of IL-1ß significantly more than CBF at the concentrations of 37.1, 18.5, and 9.3 μg/mL in A549 cells and at the concentration of 37.1 μg/mL in HEp-2 cells (Figure 8c,d). At the concentration of 37.1 μg/mL, the secretion of IL-6 in HEp-2 cells and the secretion of IFN-γ in A549 cells in the SHL group was less than those in the CBF group (Figure 8f,g). There was no significant difference between the treatment groups for IL-6 secretion in A549 cells and IFN-γ secretion in HEp-2 cells (Figure 8e,h). Taken together, these results suggest that SHL can attenuate the inflammatory effects in HAdV_3_-stimulated A549 and HEp-2 cells more than CBF.

## 4. Discussion

Herb formula or natural compound drugs have been shown to be effective in the TCM clinic. Indeed, various compounds contained in herbs or formula (mixture of herbs) are used therapeutically and play an important role in rebalancing disorders in organisms. TCM formulas are always considered multi-component and multi-target agents, which is essentially the same strategy as that during combination therapy with multi-component drugs. The ratios of bioactive ingredients affect efficacy and toxicity, as occurs with highly active antiretroviral therapy and ginaton. Therefore, this study was performed to determine a consistent dose and compounding ratio of CBF. According to the results of HPLC (Figure 1), chlorogenic acid (1.3%), forsythoside A (0.8%), and baicalein (21.6%) were the major components of SHL. CBF was a combination of the ratio of the results of HPLC.

We used a plaque reduction assay to determine the effects of SHL and CBF in both HEp-2 and A549 cells. Variation of the anti-virus activities of SHL and BCF was correlated with its working point after administration. According to our study, the antiviral ability of SHL was greater than that of CBF when the cells were treated before viral inoculation. This antiviral potency of SHL was further confirmed by its ability to inhibit viral attachment and internalization (Figure 5 and Figure 6), suggesting that the anti-viral ability of SHL is mainly a preventative effect. Therefore, longer pre-incubation times improve the anti-viral effects of the two drugs. Moreover, different anti-virus assays and different respiratory tract cell lines were tested. Compared with CBF, SHL had a high CC_50_ and inhibited virus entry and replication. Therefore, SHL is safer, more effective, and more readily available than CBF for the prevention and management of virus-induced airway injuries.

Although both SHL and CBF can disrupt virus DNA amplification and destroy virus particles, SHL treatment reduced virus DNA load in cells more than CBF. The reduction in virus DNA load was greater with SHL than CBF within or out of the two cell types, A549 and HEp-2 cells. In cell suspensions, the anti-viral effects of intervention with SHL were greater than the effects with CBF in A549 cells but not HEp-2 cells, suggesting that SHL was a more effective drug at improving the lower respiratory tract infections in virus. What is more, the sensitivity of the virus to the two cells was different; the amount of the virus released was also different; and the low levels of virus in the supernatant of the cells may be one reason for the absence of differences between the two cells.

Virus-induced secretion of cytokines, such as INF, TNF, and IL, contribute to innate immunity against viral infection. Therefore, variations in the levels of IFN-γ, TNF-α, IL-1ß, and IL-6 were compared between SHL and CBF treatment groups [23,24,25,26,27]. According to our study, both SHL and CBF attenuated the production of pro-inflammatory cytokines IL-1ß, IL-6, and TNF-α and promoted the release of INF-γ in vitro as compared to the virus control group, suggesting that SHL and CBF can suppress inflammation stimulated by virus. In addition, the regulation of inflammation markers was greater for SHL than that for CBF in both cell types. Consistent with this finding, SHL had a multi-channel anti-virus effect and protected against respiratory injury from HAdV_3_ infection better than the monomer combination.

SHL has been used as an empirical therapy to treat virus infection in the TCM clinic for many years. This study is the first to evaluate comprehensively the role of SHL as an antiviral agent against HAdV_3_. Furthermore, the antiviral activity of SHL was compared to CBF—the main compound combination in SHL—to evaluate the potential anti-virus activity of the effective parts and its composition. Parallel doses and combination ratios of these ingredients were determined and controlled by HPLC. We demonstrated that both SHL and CBF effectively inhibited HAdV_3_-induced injuries by preventing viral penetration; un-coating; mRNA translation; protein synthesis; genome replication; and virus assembly and release to counteract viral infection. SHL appeared to be very promising in terms of efficacy and toxicity. This study also provides a primary explanation for the hypothesis that multiple bioactive ingredients with multiple targets of TCM are involved in the antivirus mechanism of SHL. Although CBF was not an ideal combination of the compound ingredient, our findings are helpful for related drug discovery and merit further exploration.

## Abbreviations

The following abbreviations are used in this manuscript:

ANOVA	analysis of variance
CBF	chlorogenic acid, baicalin, forsythia glycosides A
CC50	50% cytotoxic concentrations
CCK-8	cell counting kit-8
DAD	diode array detector
DMEM	Dulbecco’s Modified Eagle’s Medium
ELISA	enzyme-linked immunosorbent assay
FBS	fetal bovine serum
HAdV	human adenovirus
HIV	human immunodeficiency virus
HPLC	high performance liquid chromatography
HSD	honestly significant difference
IC50	50% inhibitory concentration
IFN	interferon
IL	interleukin
PCR	polymerase chain reaction
qPCR	quantitative polymerase chain reaction
S.D.	standard deviation
SHL	Shuang–Huang–Lian injectable powder
TCID50	50% tissue culture infection dose
TCM	traditional Chinese medicine
TNF-α	tumor necrosis factor alpha
DNA	deoxyribonucleic acid
